# In-vivo, non-invasive detection of hyperglycemic states in animal models using mm-wave spectroscopy

**DOI:** 10.1038/srep34035

**Published:** 2016-09-27

**Authors:** Pedro Martín-Mateos, Fabian Dornuf, Blanca Duarte, Bernhard Hils, Aldo Moreno-Oyervides, Oscar Elias Bonilla-Manrique, Fernando Larcher, Viktor Krozer, Pablo Acedo

**Affiliations:** 1 Department of Electronics Technology, Universidad Carlos III de Madrid, Leganes, Madrid 28911, Spain; 2 Physics Institute, Goethe University Frankfurt am Main, 60438 Frankfurt am Main, Germany; 3 Epithelial Biomedicine Division, CIEMAT, Avenida Complutense 40, Madrid, 28040, Spain; 4 Centro de Investigación Biomédica en Red de Enfermedades Raras (CIBERER), Madrid, Spain; 5 Department of Bioengineering, Universidad Carlos III de Madrid, Leganes, Madrid 28911, Spain; 6 Instituto de Investigaciones sanitarias de la Fundación Jimenez Diaz (IIS-FJD), Madrid, Spain

## Abstract

Chronic or sustained hyperglycemia associated to diabetes mellitus leads to many medical complications, thus, it is necessary to track the evolution of patients for providing the adequate management of the disease that is required for the restoration of the carbohydrate metabolism to a normal state. In this paper, a novel monitoring approach based on mm-wave spectroscopy is comprehensively described and experimentally validated using living animal models as target. The measurement method has proved the possibility of non-invasive, *in-vivo*, detection of hyperglycemia-associated conditions in different mouse models, making possible to clearly differentiate between several hyperglycemic states.

Diabetes is nowadays a worldwide epidemic problem. Long-term diabetes complications include cardiovascular disease, nephropathy, neuropathy, and retinopathy^1^,^2^,^3^. Thus, diabetes management, meaning restoration of carbohydrate metabolism to a normal state is critical to prevent such complications. Although blood glucose content is the main parameter to evaluate the acute diabetic state and a critical factor to take action in type I diabetes^4^, other metabolites more accurately inform on the proper glycemic control over longer periods (e.g. sustained hyperglycemia). Among them, glycated hemoglobin (HbA1c) has been shown to be a reliable marker^5^,^6^,^7^.

The complexity of events leading to cellular malfunction in response to high levels of glucose is not fully understood. One of these events is the formation of advanced glycation end-products (AGEs)^8^,^9^,^10^. The elevated levels of glucose results also in the formation of covalent adducts not only with plasma proteins, such as albumin, hemoglobin or fibrinogen, but also with extracellular matrix proteins such as collagen and fibronectin^11^,^12^,^13^, through a non-enzymatic process known as glycation. Thus, persistently elevated glucose levels during sustained diabetes induce structural and functional changes in these proteins likely contributing to diabetic multi-organ malfunction. While serial measurements of HbA1c represent a standard marker for long-standing glycemic control, only few other reliable markers have been described like glycated albumin or albumin-corrected fructosamine^14^,^15^,^16^,^17^.

In the present study we show, using different long-standing diabetic mouse models, that a mm-wave spectroscopic analysis of the transmittance of the skin of the mice is capable to categorize animals undergoing sustained hyperglycemia from those under stable normolgycemic conditions. Measurements performed *in-vivo* and non-invasively, make possible to accurately differentiate between control and various hyperglycemic states, likely through the detection of AGEs not yet characterized. These results validate the measurement technique and procedure, proving the possibility of the non-invasive detection of hyperglycemia-associated conditions on living animal models. Moreover, the instrument is based on well-established and potentially low-cost electronic technology, paving the way for next generation instrumentation for diabetes monitoring and control.

## Results

### Measuring principle

**Figure 1:**
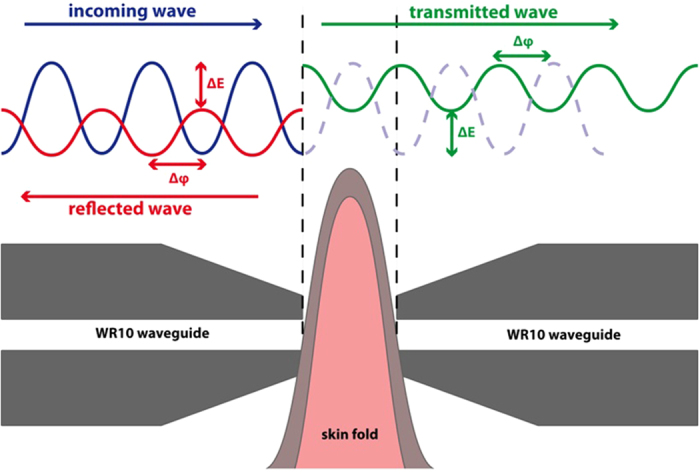
Basics of the measurement set-up.

As the basis of the proposed method for the monitoring of sustained hyperglycemia, the transmittance of a fold of the skin at the nape of the mice is measured at 25 equally spaced frequencies located within the W-band (75–110 GHz) in the mm-wave range, a block diagram of the measurement head is shown in Fig. 1 (see Methods section for further details).

Mice with different hyperglycemic states (i.e. diabetes with different degrees of severity) and from different strains (see Methods section for a comprehensive list of the animals used in the tests) were used in the experiment in order to study the sensitivity of the proposed system, not only to the level of sustained hyperglycemia, but also to several factors of influence in the measurement. Besides the use of control, and genetically obese and diabetic animals, different sustained glucose levels were forced by both diabetizing healthy mice and by the implantations of leptin pumps in obese animals. Therefore, the hyperglycemic states include control mice (of different strains), obese mice with mild and sustained diabetes, genetically diabetic mice with high and sustained diabetes, and diabetized mice with sharp and acute blood glucose increase. In addition, and as previously introduced, some obese animal models, which were made normolgycemic by the implantation of leptin pumps, have also been included in the tests.

**Figure 2:**
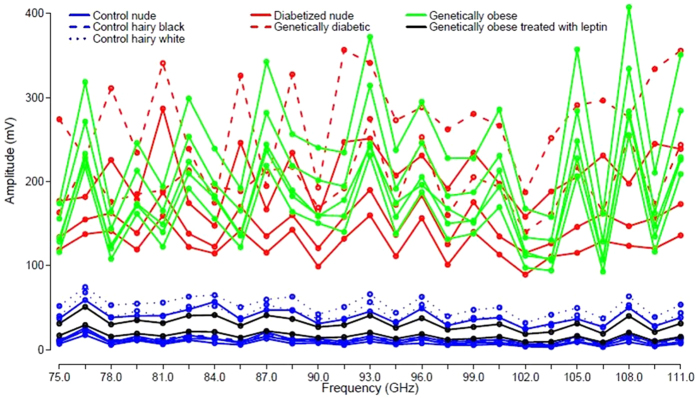
Amplitudes of the signals at the output of the receiver for the different types of mice. As the maximum standard deviation found in the measurements is below 2%, error bars have not been included.

The amplitudes of the signals transmitted through the folded skin for each animal model as a function of frequency are shown in Fig. 2. It is apparent how the transmittance of the skin of the group of animals with higher blood glucose levels is far higher than that of animals with normal glucose levels. It is clearly appreciated that the different strains of control normoglycemic animals (nude and hairy black and white mice) and the obese implanted with the leptin pump have a similar skin transmittance. On the other hand, obese and genetically and chemically diabetic mice, with a much lower absorbance, can be grouped together.

**Figure 3:**
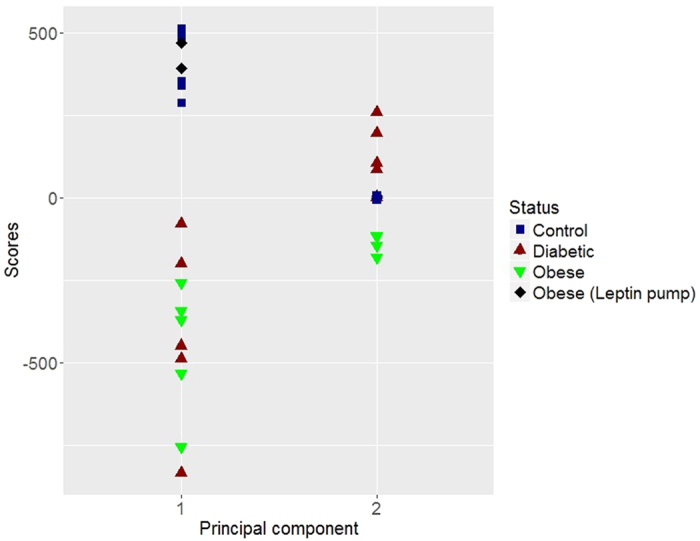
Scores of the different mice groups for the two first principal components.

Principal Component Analysis (PCA) techniques were applied to the data matrix from Fig. 2 in order to reduce the dimensionality of the data (see Methods section for further details). The scores of the two first Principal Components (PCs) that were found to explain the 94.75% and the 4.86% of the variance, respectively, (with a cumulative explained variance of 99.61% between them), are shown in Fig. 3. From this figure it is clear how the score of PC 1 (first PC), accounting for the highest variance in the measurements, can be directly used to differentiate the control animals from hyperglycemic mice. In this way, the measurement set-up together with the previously presented data analysis is capable of providing a single indicator (score of PC1) that differentiates between control and hyperglycemic mice. It is also equally worth noting the behavior of PC 2 (second PC), that differentiates between control animals (including all the mice strains detailed below) that are grouped together, obese, and diabetized and genetically diabetic mice. Another very interesting observation is that our measurement set-up is capable of detecting the changes taking place in obese mice after leptin replacement for 25 days, classifying them as control animals.

**Figure 4:**
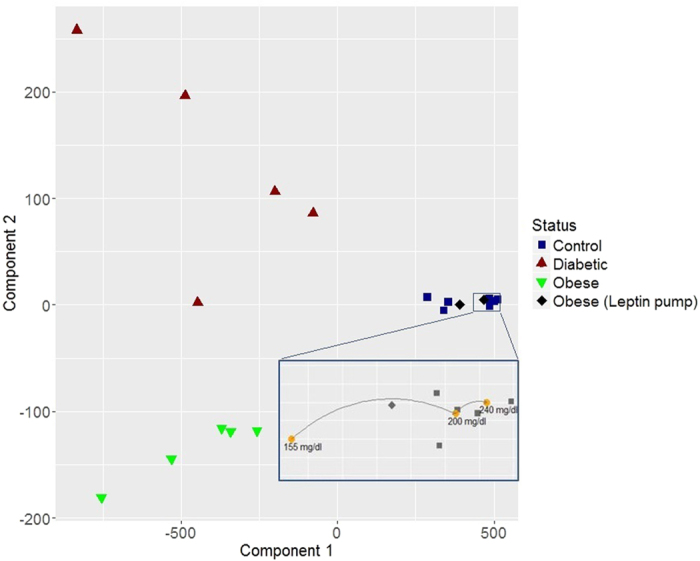
Representation of the scores for the first principal component versus the scores of principal component 2. Zoomed-in area: Results of the PCA classification for different instantaneous blood glucose levels forced on a control mouse (yellow dots).

To better illustrate the classification of hyperglycemic states, in Fig. 4, the scores of the first principal component have been represented against the scores of the second principal component. Three separated areas hence appear, one for the control and leptin pump-treated mice, a different one for the diabetic and diabetized mice, and a third one for obese animals. Therefore, this instrument it is not only capable to separate hyperglycemic from control mice, but it is also able to distinguish between different hyperglycemic states.

In order to evaluate other biological variables that may have an influence on the measurements, the thickness of the skin in the measurement area (Table 1) and the influence of the blood glucose level (Fig. 4, zoomed-in area) were studied. The control group presents, in average, a 28% thicker skin than the diabetic group mainly because of the use of nude animals, but no difference in thickness was found between the control and diabetic black hairy mice (see Methods). The thickness of the skin of the obese animals almost doubles the thickness of the diabetic, but nonetheless, they are properly classified in the corresponding group in the hyperglycemic/normoglycemic decision by the set-up presented in this paper (Fig. 3). This study indicates the robustness of the instruments to the thickness of the skin, and that the accumulation of subepidermal fat does not influence the correct classification of the hyperglycemic state.

For the study of the influence of the blood glucose level on the measurements, an additional experiment consisting on forcing a blood glucose increase on a mouse was carried out while monitoring, with the proposed sensor, the transmittance of the skin. A control animal was measured (following the same protocol than for the rest of mice) just before injecting a glucose solution; additional measurements were then taken ten and twenty minutes after the injection. Blood glucose was determined after each measurement finding an increase from 155 mg/dl to 200 mg/dl in ten minutes and up to 240 mg/dl after twenty minutes. In this way, three spectral characterizations for the same animal with an important difference in the actual blood glucose (of almost twofold) were obtained. The scores of the PCA analysis for the first and second principal components of these three measurements are shown in Fig. 4 (yellow dots in the zoomed-in area) in combination with the previous results. This test indicates that the detection system is not sensitive to the instantaneous blood glucose level found at the moment of taking the measurement, but to the effects of sustained hyperglycemia.

## Discussion

In this study, transmittance spectra from the skin of various mouse models comprising a big range of hyperglycemic states have been acquired. On one hand we obtained measurements from animals undergoing a sharp and acute increase in glucose levels leading to an extremely diabetic state of >400 mg/dL (chemically diabetized nude mice) and a highly and sustainably diabetic state with glucose >250 mg/dL (black diabetic db/db mice). The sensor provides a similar output in both situations, not finding significant differences between these two groups. Apart from them, another population of mildly and sustained diabetic with glucose levels of >150 mg/dL (black leptin deficient obese ob/ob mice) could be clearly differentiated from the first and also form a control group with normal glucose levels. At the view of the analysis of results and the extra tests that were performed, it is clear that the developed spectroscopy instrument is insensitive, not only to the thickness and fat content of the skin, but also, and much more importantly, to the actual blood glucose level at the moment of performing the measurement. The performance of the instrument remains also unaffected by skin properties such as, e.g. hairy, shaved, or nude skin. This indicates that the technique is rather robust against surface skin properties, in contrast to optical methods, which show a strong dependence on skin surface characteristics like the existence of hair or melanin content. The fact that the set-up is not directly monitoring blood glucose levels, point to other metabolites, likely AGEs affected by sustained hyperglycemia, as responsible of our measurements. Further studies will be devoted to the characterization of these altered metabolites.

Several reasons make mm-waves in the W-band a frequency region that it is very well suited for the studies described in this manuscript. First, the small dimensions of the W-band waveguides provide a small probing area and, in the same way, the small penetration into the body allows for avoiding any interference in the measurements results due to secondary effects. Other advantages are the proven sensitivity to constituents in body fluids and the high dynamic range (much higher than at THz).

In summary here a robust and reliable mm-wave spectroscopic instrument and the corresponding analysis technique for the *in-vivo*, non-invasive, detection of sustained hyperglycemia on living animal models (mice) have been presented. The tests, performed on animals of various strains and different hyperglycemic levels, prove that it is possible to clearly identify and differentiate control and diabetic mice with increasingly higher sustained blood glucose levels *in-vivo* and non-invasively. Even though most previous publications on this field target the measurement of the instantaneous blood glucose level instead of the maintained blood glucose level detected in the experiments presented in this manuscript, it is through the measurement of biomarkers related to sustained hyperglycemia like glycated hemoglobin (HbA1c) how diabetes mellitus can be more accurately monitored. The method here presented provides a much higher temporal resolution than that of HbA1c and, as the instrument is based on well-established and potentially low-cost electronic technology, our results might pave the way for the development of the next generation of instruments for diabetes control.

## Methods

### Animals and glucose level modulation treatment

Twenty mice of different strains were used in the experiment. The list includes seven nude NMRI-*Foxn1*^*nu*^/*Foxn1*^*nu*^ mice, four of them were selected as control and the other three were chemically diabetized by three intraperitoneal injections of Streptozotocin (Sigma-Aldrich, Inc., Missouri, USA) with concentrations of 0.1 mg/g, 0.1 mg/g and 0.15 mg/g during three consecutive days. Measurements were performed 15 days after the first diabetization dose was injected. Additionally, two spontaneously diabetic, black (Lepr^db^/Lepr^db^) and seven leptin deficient, obese black (Lep^ob^/Lep^ob^) mice were employed. Two of the ob/ob animals were rendered lean and normoglycemic by implantation of a 28-day-lasting micro osmotic pump (ALZET Osmotic Pumps, California, USA) containing human leptin. Measurements were taken 25 days after implanting the pump. Finally, two control black C57Bl6/J and two control albino BalbC mice were used. A detailed list of animals is shown in Table 2 for further clarification.

These animals can be classified in two different groups. The first group includes all the control and the leptin-treated animals. For those animals the blood levels of glucose remained normal during approximately the 25 days prior to the experiment. The second group, with sustained hyperglycemia for at least 12 days before the measurements, includes the chemically –induced diabetic, and the genetically diabetic db/db and ob/ob mice.

All animals were purchased from Elevage-Janvier (France) and housed individually in pathogen-free conditions at the Centro de Investigaciones Energéticas, Medioambientales, y Tecnológicas (CIEMAT) Laboratory Animals Facility (Spanish registration number 28079-21 A).

### Experimental protocol

All experimental procedures were carried out according to European and Spanish laws and regulations (European convention ETS 1 2 3, about the use and protection of vertebrate mammals used in experimentation and other scientific purposes, Directive 2010/63/UE and Spanish Law 6/2013, and R.D. 53/2013 about the protection and use of animals in scientific research). Procedures were approved by the Animal Experimentation Ethical Committee of the CIEMAT according to all external and internal bio-safety and bio-ethics guidelines, and by Spanish competent authority with registered number PROEX 176/15.

Prior to the measurement, mice were anesthetized one by one, using standard rodent anesthesia (ketamine/medetomidine). The injection was given five minutes before taking the measurement. The measurement process is as follows. The skin sample is placed between two straight cuts of a WR10 waveguide, a frequency sweep across the W-band is performed, and the amplitude and phase of the signal transmitted through the skin are continuously acquired using the mm-wave spectroscopic instrument described below. The measurement time for one sweep was around 45 seconds. Transmittance data is further processed by PCA in order to obtain clear indicators of the existence of chronic or sustained hyperglycemia and the hyperglycemic state.

**Figure 5:**
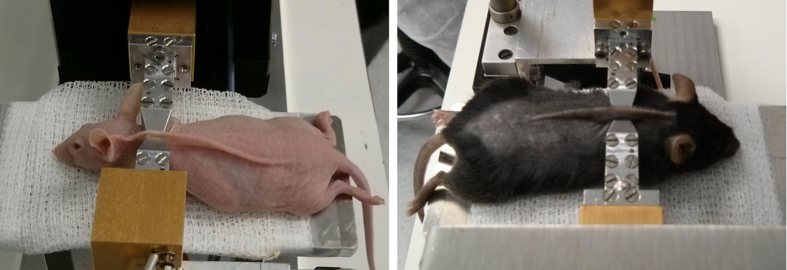
Images of the folded mouse back skin setting used to measure the transmittance of each sample for a diabetized nude (left) and a control black (right) mice.

To ensure the non-invasive operation, all measurements were carried out on a fold of the skin of the mice (see Fig. 5), not harming the animals. A separation between waveguides of 800 μm was used as it was found to be the best compromise for having the length necessary to fix the skin during measurements, and not harming the mice. The measurement area of all hairy mice was shaved to ensure the consistency of the data obtained and to avoid the influence of possible external factors on the measurements. It is important to note here that, besides shaving the hairy animals, no previous treatment of the skin, or gel, is needed to perform the measurements.

Additionally, and for each animal, the thickness of the skin in the measurement area was determined by a Mitutoyo 543–781B gauge (Mitutoyo Corp., Kanagawa, Japan). The blood glucose was tracked during the glucose injection experiment by an Accu-Chek Aviva Nano (Hoffmann-La Roche, Basilea, Switzerland).

### Non-invasive mm-wave spectroscopic instrument for hyperglycemia assessment

**Figure 6:**
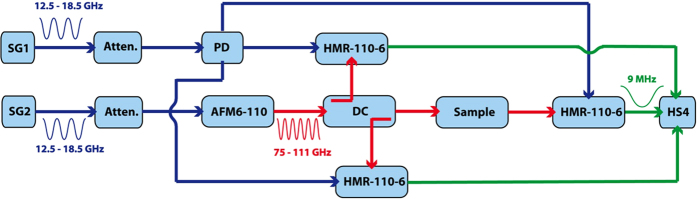
Block diagram of the measurement set-up. SG, Signal Generator; Atenn, Variable Attenuator; PD, Power Divider; AFM, Active Frequency Multiplier; DC, Directional Coupler; Sample; Skin sample (animal); HMR, W-Band Receiver; HS4, Acquisition Hardware. The blue wires represent base band signals (12.5–18.5 GHz), the red wires high frequency signals (75–111 GHz) and the green wires represent intermediate frequency signals (9 MHz).

**Figure 7:**
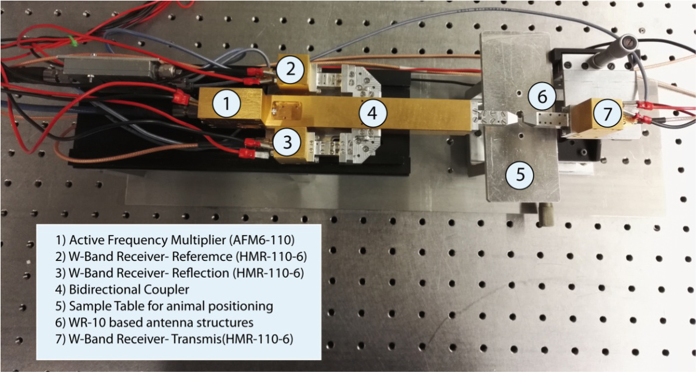
Picture of the measurement set-up.

A mm-wave spectroscopic instrument working in the W-band was used for the experiment, which is tuned from 75 to 110 GHz in steps of 1.5 GHz acquiring at every point the amplitude and phase of the signal transmitted through and reflected from the skin samples. The simplified block diagram of the set-up employed in the measurements is shown in Fig. 6 and a photograph of the instrument in Fig. 7.

An AFM6-110 Active Frequency Multiplier (Radiometer Physics GmbH, Meckenheim, Germany) with a multiplication factor of six and a frequency range from 75 to 110 GHz is employed in the generation of the high frequency signal that is directly coupled into a WR10 waveguide. A directional coupler is used together with a HMR-110-6 W-band receiver (Radiometer Physics GmbH, Meckenheim, Germany) to obtain a reference measurement of the generated signal. The folded skin under test (Fig. 5) is placed between two structures in which the WR-10 waveguides are rejuvenated to decrease the required skin fold area. Two additional HMR-110-6 receivers are employed to measure the signal transmitted through the sample and the signal reflected from the surface of the skin fold. The radio frequency and local oscillator signals needed for the generation of the mm-wave radiation (SG1 and SG2 respectively in Fig. 6) are swept between 12.5 GHz and 18.5 GHz in steps of 250 MHz using APSYN420 (AnaPico, Zurich, Switzerland) synthesizer. To avoid the saturation of the receivers, the set-up is supplemented with switchable attenuators, which adjust the input power to the frequency multiplier and the receivers at each frequency setting. For the acquisition of the signals a frequency offset of 1.5 MHz is set between the AFM6-110 Frequency Multiplier and the local oscillator frequency of the W-band receiver producing a 9 MHz intermediate frequency for the signals in the 75–110 GHz frequency range. The 9 MHz intermediate frequency output signals for the reference, reflection and transmission channels are directly sampled at 10 MHz by an acquisition card (Handyscope HS4-10, TiePie engineering, Sneek, Netherlands) and processed using LabView. The program, running in real-time and working as a multi-channel lock-in amplifier, was designed to obtain the amplitudes of the signals reaching the receivers and the phase difference between the reference signal to either the transmitted or the reflected signal.

### Data processing Principal Component Analysis

The whole set of measurements of transmittance of the skin of the mice was processed using PCA techniques^18^. The principal components are a linear combination of the original variables that create an orthogonal basis in which the original data can then be projected. The PCs are sorted so that the first component picks up the largest share of the original variability; the second component must collect the maximum possible variability not picked up by the first, and so on. The theoretical basis of the method and the data processing performed in this manuscript are described below.

We will assume that X_*nxp*_ is a data matrix which contains the measurements of *p* quantitative variables (characteristics) of a sample of size *n* (observations), in order to simplify the problem the data is centered by columns (variables). Hereinafter, we will denote 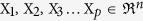 as variables and 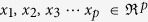 as observations. The principal components, that as presented above are linear combinations of the original variables, are represented by 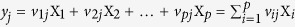, where the coefficients *v*_1_*j*, … , *v*_*pj*_ are constants known as loadings of the *j*th principal component (that basically define the direction of the component). In general, for all the components we have the relationship described by equation (1), where Y is the score matrix which contains the rotated data, V is the rotation matrix with the loadings of the principal components, and X is the data matrix centered.


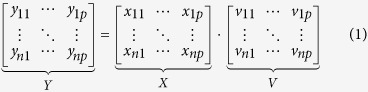


As each of the original variables in X has been centered to have zero mean, the principal components also have zero mean. For the first component *y*_1_ the variance is given by 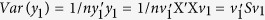, where S is the covariance matrix of X. The variance can be maximized by increasing the module of *v*_1_, hence to find a unique solution to this problem, we impose a restriction on *v*_1_, such that 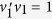. Then, we apply the Lagrange multiplier method getting, by definition, 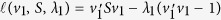 and by solving the last expression we have that *Sv*_1_ = *λ*_1_*v*_1_, which implies that *λ*_1_ is an eigenvalue of S and *v*_1_ is its corresponding eigenvector. Moreover, by multiplying 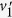 by the previous equation we get that 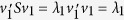, so *λ*_1_ is the variance of the first principal component, and is the greater eigenvalue of S.

As the principal components are independent, for the second principal component an additional restriction is added to the function: 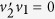, then, by applying again the Lagrange multiplier method we get 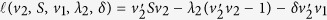. By solving the last expression (considering the restrictions above mentioned) we get *Sv*_2_ = *λ*_2_*v*_2_, which implies that *λ*_2_ is an eigenvalue of S and *v*_2_ its corresponding eigenvector, being *λ*_1_ and *λ*_2_ the greatest eigenvalues of S in decreasing order. Following the same argument, we can obtain all the principal components.

As the covariance matrix is symmetric, the principal components can be obtained from applying singular value decomposition on the covariance matrix of X, since the singular values coincide with the eigenvalues which are the variances of the principal components, and the singular vectors coincide with the eigenvectors which are the loadings of the principal components. The input to the algorithm was the data matrix composed of the number of mice observations as rows and frequency points as columns.

All the data analysis presented in this paper was performed using the PRCOMP package available in the library of the open source software R^19^.

## Additional Information

**How to cite this article**: Martín-Mateos, P. *et al.* In-vivo, non-invasive detection of hyperglycemic states in animal models using mm-wave spectroscopy. *Sci. Rep.* **6**, 34035; doi: 10.1038/srep34035 (2016).

## Figures and Tables

**Table 1:** Thickness of the skin in the measurement area for the different mice groups.

	Control	Obese	Diabetic
Average (μm)	394	605	308
Standard deviation (μm)	83	51	117

**Table 2:** List of animals employed in the tests.

Mice strain	Variation	Expected glucose level	Time with the condition	Referenced as	Number of animals
NMRI-*Foxn1*^*nu*^/*Foxn1*^*nu*^	—	100 mg/dL	1 month	Control nude	4
C57Bl6/J	—	100 mg/dL	6 months	Control hairy black	2
BalbC	—	100 mg/dL	6 months	Control hairy white	2
Lep^ob^/Lep^ob^	Normoglycemic by leptin-pump	100 mg/dL	25 days	Genetically obese treated with leptin	2
Lep^ob^/Lep^ob^	—	>150 mg/dL	6 months	Genetically obese	5
Lepr^db^/Lepr^db^	—	>250 mg/dL	6 months	Genetically diabetic	2
NMRI-*Foxn1*^*nu*^/*Foxn1*^*nu*^	Diabetized	>400 mg/dL	12 days	Diabetized nude	3
